# Diet Quality Among Older Adolescent Boys and Girls in the Southeast Asia Region

**DOI:** 10.1111/mcn.13774

**Published:** 2024-12-04

**Authors:** Alissa M. Pries, Alison Feeley, Roland Kupka

**Affiliations:** ^1^ UNICEF East Asia Pacific Regional Office Bangkok Thailand

**Keywords:** adolescents, diet indicators, diet quality, Southeast Asia

## Abstract

Adolescence is a period of tremendous physical and neurophysiological change, and today's rapidly changing food system has implications for adolescent nutritional and health outcomes. Ensuring nutritious diets during adolescence requires evidence on what is being consumed by adolescent boys and girls, however, little is known about the dietary patterns among this age group. This study assessed the prevalence of food group consumption and indicators of diet quality among adolescents in the Southeast Asia region and compared these results to the adult population. Secondary analysis of the Gallup World Poll, a population‐based survey, was performed using datasets from Cambodia, Indonesia, Laos PDR, the Philippines and Vietnam. Pooled analysis of nine diet quality indicators was conducted among all adolescents 15–19 years of age (*n* = 479), with comparison to the adult population (*n* = 4589). Various unhealthy food groups were consumed by one‐quarter to one‐half of adolescents, with a greater proportion of adolescents consuming instant noodles, sweets, processed meats and salty snacks, as compared to adults. Just over one‐third of adolescents (37.4%) consumed all five recommended food groups to meet dietary guidelines, almost two‐thirds (62.6%) consumed sweet beverages and over three‐quarters consumed unhealthy/ultra‐processed foods (76.8%). Overall indicators of diet quality showed that Southeast Asian adolescents' diets were less healthy than adults. This is one of the first studies to explore the healthy and unhealthy aspects of diets among both adolescent boys and girls across the Southeast Asia region, with results indicating that diets are not nutritionally adequate.

## Summary

- Unhealthy dietary patterns were marked among adolescents across the five Southeast Asian countries. Up to one‐half of adolescents consumed certain unhealthy food groups, including sweet drinks, instant noodles, sweets, deep‐fried foods and processed meats. Compared to adults, adolescents had a higher prevalence of indicators of unhealthy diets.
- Diet quality concerns differed by urban versus rural adolescents, with the consumption of nutritious foods less prevalent among rural adolescents and the consumption of unhealthy foods more prevalent among adolescents in urban locations.
- These unhealthy dietary patterns have concerning health and nutrition implications. Systemic‐level programme and policy actions are urgently needed to curb the rise of excess weight gain and the potentially long‐term effects of poor diets.

## Graphical abstract

This study assessed the prevalence of food group consumption and indicators of diet quality among 479 adolescents in five Southeast Asian countries and found that overall diet quality among Southeast Asian adolescents was less healthy than among adults. This is one of the first studies to explore the healthy and unhealthy aspects of diets among both adolescent boys and girls across the Southeast Asia region, with results indicating that diets are not nutritionally adequate.

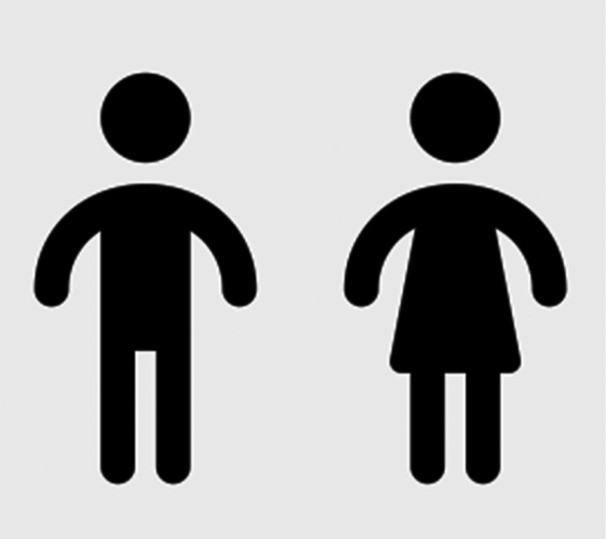

## 1 Introduction

The period of adolescence, between the ages of 10–19, is one of tremendous physical and neurophysiological change. Adolescents experience a rapid increase in growth and emotional and social development (Adedini et al. 2016; Depauw and Oxley 2018). Because of the importance physically and cognitively of this period, ensuring adequate diets and nutrition is crucial for establishing healthy eating habits that can be lifelong. Prior research has shown that adolescents may have poorer diet quality than older populations, including adults and older adults (Andrade et al. 2016), and understanding where diets among adolescents are lacking in comparison to the general adult population may be beneficial for tailoring targeted public health nutrition strategies to foster healthy eating during this critical stage of diet habit formation.

There are over 1 billion adolescents globally, with the vast majority living in low‐ and middle‐income countries (LMICs) where access to nutritious diets can be limited (UNICEF 2022). The prevalence of micronutrient deficiencies among adolescents in LMIC contexts is marked—with over 20% of boys and girls experiencing iron deficiency anaemia, vitamin A deficiency, with rates of iron deficiency slightly higher among girls and vitamin A deficiency slightly higher among boys (Christian and Smith 2018). In East and Southeast Asia, over 25% of adolescent boys and girls are underweight, with some of the lowest BMIs globally among adolescents noted in contexts like Cambodia and Vietnam (Akseer et al. 2017; NCD Risk Factor Collaboration 2020; Abarca‐Gómez et al. 2017).

Adolescents are also growing up in a rapidly changing food system. Wide availability of affordable, convenient foods and exposure to targeted marketing can encourage the consumption of ultra‐processed commercial foods, detrimentally affecting nutrition and health (Costa et al. 2018; Martins et al. 2022). Concurrent with persistent undernutrition, the prevalence of overweight has been steadily climbing in LMICs, with over 25% of adolescent girls and nearly 20% of adolescent boys living with overweight or obesity in East and Southeast Asia (Christian and Smith 2018). The emerging high prevalence of adolescent overweight and obesity in Southeast Asian countries, combined with persistent undernutrition and micronutrient deficiencies, suggests these social/physical environmental changes are having profound health consequences in LMICs.

Despite clear indications that adolescents in LMICs are suffering a triple burden of malnutrition—experiencing prevalent rates of overweight or obesity, micronutrient deficiencies and undernutrition, there is extremely limited data on the contributing consumption patterns and diet quality of adolescents in these contexts (Demmler et al. 2024; Kupka, Siekmans, and Beal 2020). Much of the existing global data on adolescent diets is derived from school‐based surveys, primarily the Global School‐Based Student Health Survey (GSHS) or national population‐based surveys among women of reproductive age, primarily the Demographic Health Surveys (DHSs). While these surveys do provide details on what sub‐populations of adolescents are consuming, they are not representative of adolescent populations. As a school‐based survey, the GSHS fails to capture data among adolescents who are not attending school and as the DHS only includes older adolescent females in its sampling it fails to cover adolescent boys. Further, there is a limit in the types of dietary assessment tools within these studies that are used among adolescent samples that restrict the ability to fully understand how healthy or unhealthy their diets are (Christian and Smith 2018). Specifically, most surveys and studies report solely on the consumption of certain food groups or report indicators of diet quality that speak only to micronutrient adequacy, such as minimum dietary diversity for women (MDD‐W) and do not include indicators of unhealthy diet quality and dietary patterns, such as consumption of unhealthy or ultra‐processed foods and beverages.

The nutrition of adolescents merits close attention, not only because of the accelerated development and establishment of future dietary and behaviour patterns during this period (Sawyer et al. 2012), but also because this age group represents the next generation of leadership and economic development. Ensuring nutritious diets during adolescence requires evidence on what is being consumed by young adults in order to identify what gaps in diets must be addressed. The aim of this study was to address this gap by building an understanding of the diet quality of older adolescent girls and boys (15–19 years of age) living in five countries in the Southeast Asia region. The specific objectives were to: (1) assess the prevalence of food group consumption, including groups of healthy and unhealthy foods; (2) assess indicators of dietary diversity and quality and (3) to compare the prevalence of consumption and diet indicators to the adult population in the Southeast Asia region.

## 2 Methods

For this study, secondary analysis of a data set provided by the Global Diet Quality Project was conducted; this data set was derived from a cross‐sectional survey of diet quality within the Gallup World Poll, a population‐based survey implemented in over 150 countries globally (Gallup, Harvard Department of Global Health and Population, and Global Alliance for Improved Nutrition (GAIN) 2024). Data from Cambodia, Indonesia, Laos PDR, the Philippines and Vietnam were used for pooled analysis of all adolescents 15–19 years of age, with data collection and sample size details summarized in Table 1. Internal ethical approval for the original data collection of the World Poll was obtained from Gallup's internal review board process.

**Table 1:** Gallup World Poll data collection and sample size for Southeast Asian countries.

Country	Data collection period	Sample size	Mode of interview
Cambodia	August to October 2021	1000	In‐person
Indonesia	July to October 2021	1062	In‐person
Laos PDR	August to December 2021	1000	In‐person
Philippines	August to October 2021	1000	Telephone
Vietnam	November to December 2021	1007	Telephone

### 2.1 Sampling Procedures

The Gallup World Poll utilized probability‐based sampling to achieve nationally representative samples of populations aged 15 years and older, with sample sizes of around 1000 persons per country. Interviews were conducted either via telephone or in‐person, depending on the country context, with in‐person interviews conducted in Cambodia, Indonesia and Laos PDR and telephone interviews conducted in the Philippines and Vietnam. Sampling procedures differed depending on the mode of interview. Where telephone interviews were conducted, participants were sampled either via random‐digit dialling or from a nationally representative list of phone numbers. Across varying days of the week and times of the day, interviewers called the phone numbers at least five times to reach a participant. For countries where in‐person interviews were conducted, three stages were used for sampling. The first stage involved stratification, whereby one of the following three approaches was used depending on availability of population data: (1) where recent, reliable population data was available, a single or multi‐stage cluster design was used with primary sampling units (PSU) selected based on probability proportional to population size (PPS) consisting of 100–125 clusters with 8–10 interviews designated per cluster; (2) where limited population data was available, a stratified multi‐stage cluster design was used, with PSU selected based on PPS at the state/province/district level and simple random sampling used in subsequent stages and 100–125 clusters assigned or (3) where population data was only available across broad geographies, a stratified single‐stage cluster design was used, with wards/villages selected using simple random sampling to allocate 100–125 clusters across the country. In the second stage of sampling, households within each cluster were randomly selected using standardized procedures. The respondent selection was the third stage of sampling, where the interviewer listed all household members 15 years of age or older and one was randomly selected for interview.

### 2.2 Data Collection Procedures

Telephone interviews were conducted across approximately 30 min and in‐person interviews across 1 h. All interviewers were provided in‐depth training based on a standardized training manual developed by Gallup, covering interviewing techniques and recruitment selection for in‐person interviews. Quality assurance visits were made to a random selection of both in‐person and telephone interviews to ensure consistency and proper execution of interviewing techniques and questionnaire administration.

### 2.3 Data Collection Tool

Questionnaires for the Gallup World Poll collect data on the basic demographics of respondents, with this study utilizing data captured on respondents' age, gender, educational attainment, residence and income. In addition, a module was included to capture data on the diet quality of respondents—the Diet Quality Questionnaire (DQQ). The DQQ is a standardized tool intended to allow for rapid assessment of diet quality at the population level (Uyar et al. 2023). The DQQ contains questions regarding the consumption of 29 food groups in the previous 24 h. As the specific foods/beverages consumed within each of these food groups will vary across geographies, the DQQ has been adapted for over 120 countries, with the inclusion of culturally relevant sentinel foods for each question. DQQ questionnaires for each of the five countries included in this study are available at https://www.dietquality.org/tools. The questionnaires used in the Gallup World Poll are translated into the primary language of each country where implemented, with back‐translation or independent review of the translation conducted to ensure accuracy and clarity.

### 2.4 Data Analysis

Respondent age in years was used to categorize the five‐country sample into two age groups: adolescents (15–19 years of age) and adults (20+ years of age). Prevalence of consumption of each of the 29 food groups measured by the DQQ was calculated, with the following food groups categorized as unhealthy foods to limit based on global recommendations (Global Diet Quality Project 2022; Herforth et al. 2019): processed meats, baked‐grain based sweets, other sweets, salty snacks, instant noodles, deep‐fried foods, fast foods, sweet tea/coffee/cocoa, fruit juice/fruit‐flavoured drinks and soft drinks and energy/sports drinks.

Based on population‐level indicators recommended for use with the DQQ (Global Diet Quality Project 2023), a suite of nine indicators of healthy and unhealthy diet quality was assessed in this study. This included: (1) food group diversity scores (FGDS), a continuous score based on consumption of up to 10 recommended food groups (FAO and FHI 360 2016); (2) MDD‐W, a proxy indicator for micronutrient adequacy among women of reproductive age (15–49 years) (FAO and FHI 360 2016); (3) All‐5, an indicator of diets including five food groups commonly recommended for daily consumption in national food‐based dietary guidelines (Global Diet Quality Project 2023); (4) NCD‐Protect and (5) NCD‐Risk (previously referred to as GDR‐Healthy and GDR‐Limit), with NCD‐Protect serving as a proxy indicator of dietary behaviours that are protective against NCDs, including daily consumption of at least 400 g of fruits and vegetables per day, whole grains/pulses/nuts/seeds and at least 25 g of fibre and NCD‐Risk serving as a proxy indicator of dietary behaviours that are risk factors for NCDs, including consuming more than 10% of total energy from free sugars, more than 10% of total energy from saturated fat and more than 30% from total fat, more than 5 g of salt per day, consumption of processed meat and consumption of more than 350–500 g per week of red meat and consumption of ultra‐processed foods (Herforth et al. 2020); (6) Global Dietary Recommendations (GDR) score, a score based on NCD‐Protect and NCD‐Risk (Herforth et al. 2020), (7) consumption of zero fruit/vegetable (WHO and UNICEF 2021), (8) consumption of any sweet beverage (WHO and UNICEF 2021) and (9) consumption of any unhealthy/ultra‐processed food (WHO and UNICEF 2021). Details of how these indicators were calculated are provided in Table 2. Data were analysed using Stata (version 18.0). Descriptive statistics were calculated and summarized using proportions with frequencies and medians with interquartile ranges. Mann–Whitney *U* tests were used to test differences in medians and cluster‐adjusted Pearson's *χ* ^2^ tests used to test differences in proportions between adolescents and adults. Significance was set at *p* < 0.05.

**Table 2:** Indicators used to assess diet quality among adolescents 15–19 years of age in Southeast Asia.

Indicator	Definition
Food group diversity score (FGDS)	FGDS was calculated as a continuous score (range 0–10) based on consumption of 10 food groups in the previous day: grains/tubers, pulses/legumes, nuts/seeds, dairy, meat/poultry/fish, eggs, dark green leafy vegetables, other vitamin‐A rich fruits/vegetables, other vegetables, other fruits.
Minimum dietary diversity among women (MDD‐W)	MDD‐W was calculated as the proportion of female adolescents who achieved a FGDS of ≥ 5.
All‐5	All‐5 was calculated as the proportion of adolescents consuming all five food groups commonly recommended for daily consumption in national food‐based dietary guidelines. These five food groups include: starchy staples (grains and tubers), vegetables, fruits, pulses/nuts/seeds and animal‐source foods (meat, fish, eggs, dairy).
NCD‐Protect	NCD‐Protect was calculated as a continuous score ranging from 0 to 9, with a point allocated for consumption of each of the following food groups: whole grains, pulses, nuts/seeds, vitamin A‐rich orange vegetables, dark green leafy vegetables, other vegetables, vitamin A‐rich fruits, citrus fruits, other fruits. A higher score is indicative of a greater likelihood of meeting global dietary recommendations.
NCD‐Risk	NCD‐Risk was calculated as a continuous score from 0 to 9, with 1 point allocated for consumption of each of the following food groups (except for processed meat, which is weighted by allocation of 2 points): soft drinks, baked/grain‐based sweets, other sweets, processed meat, unprocessed red meat, deep‐fried foods, fast food/instant noodles, packaged ultra‐processed salty snacks.
Global Dietary Recommendations (GDR) score	GDR score was calculated as NCD‐Protect—NCD‐Risk + 9, providing a continuous variable with a range of 0–18. A higher GDR score is indicative that the dietary behaviours of a population are more likely to meet global dietary recommendations.
Zero fruit/vegetable consumption	Zero fruit/vegetable consumption was calculated as the proportion of adolescents that did not consume any type of fruit or vegetables in the previous day.
Sweet beverage consumption	Sweet beverage consumption was calculated as the proportion of adolescents who consumed soft/energy/sports drinks, fruit juice/drinks or sweetened tea/milk/coffee in the previous day.
Unhealthy/ultra‐processed food consumption	Consumption of unhealthy/ultra‐processed foods was calculated as the proportion of adolescents who consumed baked/grain‐based sweets, other sweets, packaged ultra‐processed salty snacks, instant noodles, deep‐fried foods or fast food in the previous day.

## 3 Results

A total of 5069 participants were included in the sample across the five Southeast Asian countries. Of these, nearly 10% (9.5%, *n* = 479) were adolescents 15–19 years of age. Sociodemographic details of the sample by age groups are detailed in Table 3. Over one‐third of adolescents (34.5%) had completed primary level education or less, while 39.5% were in the top two wealthiest income quintiles. The proportion of adolescents from each of the five Southeast Asia countries ranges from just under one‐fifth to over one‐fourth, with the exception of Vietnam, which contributed 6.9% of adolescents in the overall sample. While sociodemographics among the adolescents and adults were generally similar, a greater proportion of adolescents resided in rural areas as compared to adults (63.9% vs. 55.2%, *p* = 0.026).

**Table 3:** Sociodemographic information, by age group.

	Adolescents: 15–19 years (*n* = 479)	Adults: 20+ years (*n* = 4590)	Significance test result^a^
Gender, female	60.3 (289)	57.8 (2653)	*χ* ^2^ (1, *N* = 5068) = 0.3, *p* = 0.725
Completed primary education or less^b^	34.5 (165)	40.8 (1862)	*χ* ^2^ (1, *N* = 5068) = 0.3, *p* = 0.731
Residence, rural^c^	63.9 (306)	55.2 (2531)	*χ* ^2^ (1, *N* = 5068) = 10.8, *p* = 0.026
Country			
Cambodia	18.8 (90)	19.8 (910)	*χ* ^2^ (4, *N* = 5068) = 41.5, *p* = 0.001
Indonesia	24.0 (115)	20.6 (947)	
Laos	22.8 (109)	19.4 (891)	
Philippines	27.6 (132)	18.9 (868)	
Vietnam	6.9 (33)	21.2 (973)	
Wealth quintile			
Poorest quintile	21.3 (102)	18.0 (828)	*χ* ^2^ (4, *N* = 5068) = 11.9, *p* = 0.246
Second quintile	17.3 (83)	17.5 (802)	
Third quintile	21.9 (105)	18.6 (852)	
Fourth quintile	20.5 (98)	20.3 (931)	
Wealthiest quintile	19.0 (91)	25.6 (1176)	

^a^ Proportions compared using cluster‐adjusted Pearson's *χ* ^2^ tests.

^b^ Question response was refused/unknown by one adolescent and 20 adults.

^c^ Question response was unknown by four adults.

### 3.1 Food Group Consumption Among Adolescents Versus Adults

Consumption prevalence rates for the 29 food groups measured by the DQQ are presented in Table 4. The five most commonly consumed food groups among adolescents were grain‐based staple foods (97.3%), other vegetables (71.4%), dark green leafy vegetables (64.5%), eggs (64.1%) and other fruits (60.5%), while the five least commonly consumed groups included fast foods (4.6%), yoghurt (6.7%), cheese (10.8%), nuts/seeds (18.2%) and whole grains (22.1%). The majority of food groups to limit in diets were consumed by approximately one‐quarter to one‐half of adolescents, with instant noodles (44.5%), other sweets (42.0%), baked/grain‐based sweets (41.8%) and sweet tea/coffee/cocoa (41.5%) the most commonly consumed. As compared to the adult population, a greater proportion of adolescents consumed several food groups to limit, including instant noodles (44.3% vs. 35.2%, *p* = 0.044), other sweets (42.0% vs. 26.6%, *p* < 0.001), processed meats (33.4% vs. 22.2%, *p* = 0.004) and salty snacks (26.9% vs. 14.0%, *p* < 0.001), while a greater proportion of adults consumed several micronutrient‐rich food groups, including dark green leafy vegetables (70.5% vs. 64.5%, *p* = 0.038) and citrus fruits (34.1% vs. 23.6%, *p* = 0.009).

**Table 4:** Food group consumption in the previous 24 h, by age group.^a^

	Adolescents: 15–19 years (*n* = 479)	Adults: 20+ years (*n* = 4590)	Significance test result^a^
Foods to promote			
Grain‐based staple foods	97.3 (466)	97.4 (4468)	*χ* ^2^ (1, *N* = 5068) = 0.8, *p* = 0.522
Whole grains	22.1 (106)	18.2 (836)	*χ* ^2^ (1, *N* = 5068) = 2.1, *p* = 0.313
Roots and tubers	32.8 (157)	31.8 (1458)	*χ* ^2^ (1, *N* = 5068) = 0.3, *p* = 0.717
Pulses/legumes	41.3 (198)	41.3 (1893)	*χ* ^2^ (1, *N* = 5068) = 2.3, *p* = 0.334
Nuts and seeds	18.2 (87)	21.2 (971)	*χ* ^2^ (1, *N* = 5068) = 2.7, *p* = 0.252
Vitamin A‐rich orange vegetables	43.6 (209)	44.4 (2036)	*χ* ^2^ (1, *N* = 5068) = 2.8, *p* = 0.254
Dark green leafy vegetables	64.5 (309)	70.5 (3236)	*χ* ^2^ (1, *N* = 5068) = 10.7, *p* = 0.038
Other vegetables	71.4 (342)	75.4 (3462)	*χ* ^2^ (1, *N* = 5068) = 7.1, *p* = 0.079
Vitamin A‐rich fruits	29.9 (143)	29.8 (1365)	*χ* ^2^ (1, *N* = 5068) = 1.3, *p* = 0.445
Citrus fruits	23.6 (113)	34.1 (1563)	*χ* ^2^ (1, *N* = 5068) = 17.4, *p* = 0.009
Other fruits	60.5 (290)	59.0 (2706)	*χ* ^2^ (1, *N* = 5068) = 0.6, *p* = 0.616
Eggs	64.1 (307)	61.6 (2826)	*χ* ^2^ (1, *N* = 5068) = 3.2, *p* = 0.262
Cheese	10.8 (42)	8.2 (303)	*χ* ^2^ (1, *N* = 5068) = 2.7, *p* = 0.246
Yogurt	6.7 (32)	9.7 (443)	*χ* ^2^ (1, *N* = 5068) = 5.9, *p* = 0.085
Milk	25.3 (121)	23.2 (1065)	*χ* ^2^ (1, *N* = 5068) = 0.6, *p* = 0.616
Poultry	48.4 (232)	45.1 (2071)	*χ* ^2^ (1, *N* = 5068) = 0.7, *p* = 0.577
Fish and seafood	60.3 (289)	63.3 (2906)	*χ* ^2^ (1, *N* = 5068) = 1.4, *p* = 0.386
Foods to limit			
Unprocessed red meat (ruminants)	22.8 (109)	24.4 (1120)	*χ* ^2^ (1, *N* = 5068) = 2.0, *p* = 0.305
Unprocessed red meat (non‐ruminants)	34.0 (163)	43.1 (1978)	*χ* ^2^ (1, *N* = 5068) = 4.3, *p* = 0.173
Processed meats	33.4 (160)	22.2 (1017)	*χ* ^2^ (1, *N* = 5068) = 17.0, *p* = 0.004
Baked/grain‐based sweets	41.8 (200)	37.5 (1722)	*χ* ^2^ (1, *N* = 5068) = 6.2, *p* = 0.087
Other sweets	42.0 (201)	26.6 (1221)	*χ* ^2^ (1, *N* = 5068) = 62.2, *p* < 0.001
Salty snacks	26.9 (129)	14.0 (640)	*χ* ^2^ (1, *N* = 5068) = 54.2, *p* < 0.001
Instant noodles	44.3 (212)	35.2 (1617)	*χ* ^2^ (1, *N* = 5068) = 9.0, *p* = 0.044
Deep‐fried foods	33.2 (159)	28.7 (1319)	*χ* ^2^ (1, *N* = 5068) = 4.2, *p* = 0.194
Fast foods	4.6 (22)	4.9 (224)	*χ* ^2^ (1, *N* = 5068) = 1.2, *p* = 0.453
Sweet tea/coffee/cocoa	41.5 (199)	43.7 (2004)	*χ* ^2^ (1, *N* = 5068) = 1.4, *p* = 0.449
Fruit juice/fruit‐flavoured drinks	25.9 (124)	21.6 (993)	*χ* ^2^ (1, *N* = 5068) = 5.4, *p* = 0.151
Soft drinks and energy/sports drinks	29.4 (141)	25.7 (1178)	*χ* ^2^ (1, *N* = 5068) = 1.5, *p* = 0.416

^a^ Proportions compared using cluster‐adjusted Pearson's *χ* ^2^ tests.

### 3.2 Indicators of Healthy Diets

Indicators of diet quality among adolescents as compared to the adult population in the five Southeast Asia countries are presented in Table 5. Out of a possible score of 10, the median FGDS was six food groups, while the median score out of 9 for NCD‐Protect was 4. Just over one‐third of adolescents (37.4%) achieved the All‐5 indicator. Among female adolescents, 79.6% met the recommendation for minimum dietary diversity. Indicators of healthy diets among adolescents were similar when compared to those of adults. Disaggregation of diet quality indicators among adolescents by their gender and residence are presented in Tables S1 and S2. There was no significant difference in any of the healthy diet indicators when comparing adolescent boys and girls, however, differences in diet quality were observed when comparing adolescents living in urban versus rural areas. A greater proportion of urban adolescents achieved the indicators of All‐5 compared to rural adolescents (43.4% vs. 34.0%, *p* = 0.063), with this difference likely driven by a greater consumption of the pulse/nut/seed food group among urban adolescents.

**Table 5:** Indicators of diet quality, by age group.

	Adolescents: 15–19 years (*n* = 479)	Adults: 20+ years (*n* = 4590)	Significance test result^a^
Food group diversity score	6 [5–8]	6 [5–8]	*p* = 0.261
Minimum dietary diversity—women^b^	79.6 (230)	78.6 (1627)	*χ* ^2^ (1, *N* = 5068) = 0.05, *p* = 0.875
All‐5	37.4 (179)	38.6 (1773)	*χ* ^2^ (1, *N* = 5068) = 1.8, *p* = 0.362
At least one vegetable	87.3 (418)	90.4 (4146)	*χ* ^2^ (1, *N* = 5068) = 1.9, *p* = 0.394
At least one fruit	72.4 (347)	73.4 (3390)	*χ* ^2^ (1, *N* = 5068) = 5.3, *p* = 0.145
At least one pulse/nut/seed	48.0 (230)	49.6 (2276)	*χ* ^2^ (1, *N* = 5068) = 2.5, *p* = 0.327
At least one animal‐source food	98.1 (470)	96.5 (4426)	*χ* ^2^ (1, *N* = 5068) = 7.5, *p* = 0.112
At least one starchy staple food	98.5 (472)	98.5 (4519)	*χ* ^2^ (1, *N* = 5068) = 0.02, *p* = 0.933
NCD‐protect score	4 [2–5]	4 [2–5]	*p* = 0.039
NCD‐risk score	3 [2–5]	2 [1–4]	*p* < 0.001
Global dietary recommendations score	9 [8–11]	10 [9–12]	*p* < 0.001
Zero fruit/vegetable consumption	0.0 (0)	0.0 (0)	—
Sweet beverage consumption	62.6 (300)	62.7 (2877)	*χ* ^2^ (1, *N* = 5068) = 0.05, *p* = 0.891
Unhealthy/ultra‐processed food consumption	76.8 (368)	69.5 (3188)	*χ* ^2^ (1, *N* = 5068) = 12.5, *p* = 0.010

^a^ Medians compared using Mann–Whitney *U* tests and proportions compared using cluster‐adjusted Pearson's *χ* ^2^ tests.

^b^ Among women of reproductive age (15–49 years): adolescents *n* = 289; adults *n* = 2070.

### 3.3 Indicators of Unhealthy Diets

Review of indicators of unhealthy diets revealed areas of concern for adolescents. While none of the adolescents consumed zero fruits/vegetables the previous day, almost two‐thirds (62.6%) had consumed a sweet beverage and over three‐quarters had consumed an unhealthy/ultra‐processed food (76.8%). Compared to the adult population, indicators of positive diet quality were similar, however, adolescents had a higher prevalence of unhealthy/ultra‐processed food consumption (76.8% vs. 69.5%, *p* = 0.010), a higher median NCD‐Risk score (3 vs. 2, *p* < 0.001) and a lower overall GDR score (9 vs. 10, *p* < 0.001), indicating their diets were less healthy than adults in the Southeast Asia region.

There were no significant differences in any of the unhealthy diet indicators when comparing adolescent boys and girls, however, differences were noted when comparing urban and rural adolescent populations. Consumption of unhealthy/ultra‐processed foods was significantly higher among urban as compared to rural adolescents (86.1% vs. 71.6%, *p* = 0.005) and the overall median GDR score among urban adolescents was lower than rural adolescents (9 vs. 10, *p* = 0.002).

## 4 Discussion

Nutritious diets during the adolescent period are critical to facilitate rapid physical and emotional development, however, understanding of diet quality among adolescent boys and girls in LMIC is limited. This is one of the first studies to explore the healthy and unhealthy aspects of diets among both adolescent boys and girls across the Southeast Asia region, with results indicating that diets are not nutritionally adequate. Many adolescents did not meet dietary recommendations that would ensure dietary adequacy. Furthermore, diets were marked by several unhealthy dietary practices—including consumption of processed meat, sweet beverages and unhealthy/ultra‐processed foods, with adolescent diets less healthy than the general adult population.

Consumption of foods to promote versus foods to limit was mixed among the adolescents in this study. While consumption of some nutritious food groups was relatively high, consumption of other key nutrient‐rich foods was low and up to one‐half of adolescents consumed certain unhealthy food groups that should be limited. Prior research has also noted limited consumption of nutritious food groups and high prevalence of consumption of certain unhealthy foods. A 2018 systematic review of diets among adolescent girls in LMIC noted high daily consumption of meat, poultry and fish (83%), but concerningly high rates of daily unhealthy food consumption, with 100% of girls consuming sweet and salty snack foods (Keats et al. 2018). A 2020 study, using secondary data from the GSHS identified that 29%–46% of adolescents 12–15 years of age in Cambodia, Indonesia, Laos, the Philippines and Vietnam consumed soft drinks at least once per day and 74%–90% consumed fewer than five servings per day of fruit or vegetables (Fan and Zhang 2020).

The dietary patterns of unhealthy food consumption reported by Southeast Asian adolescent boys and girls have concerning implications for the health and nutrition of this population. As many LMICs are experiencing a nutrition transition, the prevalence rates of overweight and obesity among adolescents now surpass those of underweight in many countries, following a trend previously observed in many high‐income contexts (Akseer et al. 2017). A recent pooled analysis of nutritional status indicators among children 5–19 years of age in the Southeast Asia region, including Indonesia, predicted substantial decreases in thinness and underweight between 2000 and 2030, but anticipated increases in overweight—from 6% to 17%—and obesity—from 3% to 10% (Rahman et al. 2023). The observed unhealthy dietary patterns among adolescents in this present study in five Southeast Asian countries correlate to increased risks of non‐communicable diseases (NCDs) (Budreviciute et al. 2020), which have deleterious consequences in a region already experiencing one of the steepest increases in NCD (Fritz and Fromell 2022).

Results from this study indicate that adolescents in the five Southeast Asian countries included potentially have sub‐standard diet quality and limited nutrient adequacy, however, the results of different indicators were not aligned as expected. While only one‐third of 15‐ to 19‐year‐olds consumed the All‐5 food groups that are commonly recommended in national dietary guidelines and global recommendations, the majority of adolescent girls in this study met MDD‐W, which serves as a proxy indicator of dietary adequacy for women of reproductive age. The achievement of MDD‐W among adolescent girls in this study was markedly higher than the rate noted in other population‐based surveys in the region, with MDD‐W achieved among only 53% of 15‐ to 19‐year‐olds in Cambodia's 2021–2022 DHS and 61% in the Philippines' 2022 DHS (National Institute of Statistics NIS Cambodia, Ministry of Health MoH Cambodia, and ICF 2023). This discrepancy may be partly explained by the different tools used to measure MDD‐W, with the DQQ capturing a larger number of food group questions that could result in greater recall of some foods compared to the DHS questionnaire. While consumption of certain micronutrient‐rich food categories was prevalent, the DQQ tool provides no details on quantities consumed. Prior research in the region has shown that while prevalence of food group consumption may be reported, the quantities consumed are often below those necessary to achieve dietary requirements. Secondary analysis of Indonesia's Individual Food Consumption Survey found that while 95% and 29% of adolescents 13–18 years of age reported consuming vegetables and fruits, respectively, 98% consumed inadequate amounts below WHO recommendations for fruit and vegetable intake (Hermina and Prihatini 2016). Furthermore, a school‐based survey among Indonesian adolescents found that 76% consumed less than four servings of fruits and vegetables per day (Widianto, Mulyono, and Fitriyani 2017). While the present study does provide results on proxy indicators of diet quality among adolescents, given that micronutrient deficiencies are a concern in the Southeast Asia region (Roos et al. 2019), further research that is able to quantify dietary adequacy is needed.

Findings from this analysis show that there is a critical need to address adolescent diets in the region, with programme and policy actions urgently needed to curb the rise of excess weight gain and the potentially long‐term effects of poor diets on optimal health outcomes. In addition to physical changes, adolescents are also undergoing social and emotional development, seeking both independence and societal approval, which can make them vulnerable to commercial exploitation and negative social influences (Neufeld et al. 2022). While lasting dietary habits can be formed during this time, this period of life also provides a unique opportunity for targeted interventions to positively influence lifestyle behaviours that can have lifelong benefits (Patton et al. 2022).

Systemic‐level interventions have been identified that can drive improved adolescent nutrition. The commercial and ecological drivers of food behaviours can be addressed through policy measures, such as marketing restrictions of unhealthy foods and beverages, and fiscal policies on unhealthy foods and beverages to encourage the consumption of healthier foods (Hargreaves et al. 2022). However, the impact of these policy measures on shifting purchasing and consumption behaviours among adolescents specifically is not well understood, and further research is needed to address this evidence gap. School‐based interventions, such as the provision of healthy meals, building nutrition into school curriculums and promoting healthy school food environments, are recommended programmatic actions to target adolescents within the education system (Hargreaves et al. 2022). A recent study among urban Laotian adolescents noted that the inclusion of nutrition information in lessons resulted in a protective effect, with students who did not receive this information having a 2.1 higher risk of being overweight or obese (Ivanovitch, Keolangsy, and Homkham 2020). While adolescent nutrition programming can often be focused on girls, findings from this Southeast Asia study found that diet quality was equally concerning among boys and girls and so targeting should ensure that both genders are reached and measured. Further, while consumption of recommended food groups fared slightly better among urban adolescents as compared to rural, consumption of unhealthy food groups and poor diet quality indicators were more prevalent among urban dwellers, indicating that national‐level programmes are needed but targeting may be different based on location. While the data set used for this secondary analysis did not capture information on where foods were purchased or consumed, future research on these aspects as they relate to overall diet quality would be extremely useful for the tailoring of interventions to address unhealthy diets among boys and girls.

This analysis is one of the first studies to look at a range of diet quality indicators among both adolescent girls and boys across the Southeast Asia region, expanding beyond large‐scale surveys like the DHS that only capture adolescent girls or the GSHS which is limited to school‐attending adolescents. However, there are several limitations to be noted. First, as the analysis relied on a secondary data set intended to be representative of adult populations at the national level, the sample size of adolescents at the national level is not representative and, therefore, national‐level analysis is not possible. Given the dearth of dietary data available for adolescent boys and girls, there is a need for adequate sampling design in national population‐based surveys to allow for such disaggregated analysis. Second, the sample for the secondary data set only included persons 15 years of age and older, therefore, this analysis was not able to include younger adolescents 10–14 years of age whose diets may differ (Christian and Smith 2018). Third, as consumption data were only collected at the food group level and quantities of intake were not measured, the analysis is not able to estimate the actual dietary adequacy of the adolescents but only proxy indicators of diet quality. Further validation of the DQQ for adolescent dietary adequacy, including among younger adolescents, is needed. While this study does provide insights into diet quality of adolescents in the region, further research using a tool like the DQQ among nationally representative samples of both younger and older adolescents 10‐19 years of age is needed, in addition to quantitative dietary assessments of sub‐samples to estimate actual nutrient adequacy in these diets.

## Author Contributions

A.M.P. and R.K. designed the study. A.M.P. analyzed the data and wrote the manuscript. All authors provided critical review and approved the final manuscript.

## Conflicts of Interest

The authors declare no conflicts of interest.

## Supporting information

Supporting information.

Supporting information.

## Data Availability

The data that support the findings of this study are available from Diet Quality Project. Restrictions apply to the availability of these data, which were used under license for this study. Data are available from https://www.dietquality.org/ with the permission of Diet Quality Project.
