# Supplementary material for: Diet Quality Among Older Adolescent Boys and Girls in the Southeast Asia Region
Source: Matern Child Nutr. 2024 Dec 4;21(2):e13774. doi: 10.1111/mcn.13774 (PMC11956072; doi:10.1111/mcn.13774)
Supplement: Supplementary file 1 — Supporting information. [file MCN-21-e13774-s001.docx]

| **Supplemental Table 1. Indicators of diet quality among adolescents, by gender** | | | |
| --- | --- | --- | --- |
|  | Adolescent boys  (n=190) | Adolescent girls  (n=289) | Significance test result^1^ |
| Food group diversity score | 6 [4 – 8] | 6 [5 – 8] | p=0.309 |
| All -5 | 33.7 (64) | 39.8 (115) | χ^2^ (1, N=479)=3.3, p=0.180 |
| At least one vegetable | 84.7 (161) | 88.9 (257) | χ^2^ (1, N=479)=3.1, p=0.174 |
| At least one fruit | 69.0 (131) | 74.7 (216) | χ^2^ (1, N=479)=7.3, p=0.042 |
| At least one pulse/nut/seed | 43.2 (82) | 51.2 (148) | χ^2^ (1, N=479)=4.7, p=0.124 |
| At least one animal-source food | 99.0 (188) | 97.6 (282) | χ^2^ (1, N=479)=0.01, p=0.952 |
| At least one starchy staple food | 98.4 (187) | 98.6 (285) | χ^2^ (1, N=479)=0.1, p=0.840 |
| NCD-protect score | 3 [2 – 5] | 4 [2 – 5] | p=0.110 |
| NCD-risk score | 3 [1 – 5] | 3 [2 – 5] | p=0.097 |
| Global Dietary Recommendations score | 10 [8 – 11] | 9 [8 – 11] | p=0.453 |
| Zero fruit/vegetable consumption | 0.0 (0) | 0.0 (0) | -- |
| Sweet beverage consumption | 64.2 (122) | 61.6 (178) | χ^2^ (1, N=479)=0.3, p=0.684 |
| Unhealthy/ultra processed food consumption | 76.3 (145) | 77.2 (223) | χ^2^ (1, N=479)=0.4, p=0.646 |
| ^1^Medians compared using Mann-Whitney U tests and proportions compared using cluster-adjusted Pearson's χ2 tests. | | | |
