# Supplementary material for: Diet Quality Among Older Adolescent Boys and Girls in the Southeast Asia Region
Source: Matern Child Nutr. 2024 Dec 4;21(2):e13774. doi: 10.1111/mcn.13774 (PMC11956072; doi:10.1111/mcn.13774)
Supplement: Supplementary file 2 — Supporting information. [file MCN-21-e13774-s002.docx]

| **Supplemental Table 2. Indicators of diet quality among adolescents, by residence** | | | |
| --- | --- | --- | --- |
|  | Urban residing adolescents  (n=173) | Rural residing adolescents  (n=306) | Significance test result^1^ |
| Food group diversity score | 6 [5 – 8] | 6 [4 – 8] | p=0.150 |
| Minimum dietary diversity – women^2^ | 82.0 (82) | 78.3 (148) | χ^2^ (1, N=479)=0.1, p=0.852 |
| All -5 | 43.4 (75) | 34.0 (104) | χ^2^ (1, N=479)=0.1, p=0.063 |
| At least one vegetable | 86.1 (149) | 87.9 (269) | χ^2^ (1, N=479)=2.6, p=0.207 |
| At least one fruit | 72.3 (125) | 72.6 (222) | χ^2^ (1, N=479)=0.02, p=0.917 |
| At least one pulse/nut/seed | 54.3 (94) | 44.4 (136) | χ^2^ (1, N=479)=5.5, p=0.092 |
| At least one animal-source food | 98.8 (171) | 97.7 (299) | χ^2^ (1, N=479)=1.1, p=0.351 |
| At least one starchy staple food | 98.3 (170) | 98.7 (302) | χ^2^ (1, N=479)=4.9, p=0.023 |
| NCD-protect score | 3 [2 – 5] | 4 [2 – 5] | p=0.646 |
| NCD-risk score | 3 [2 – 5] | 3 [1 – 5] | p=0.001 |
| Global Dietary Recommendations score | 9 [7 – 10] | 10 [8 – 11] | p=0.002 |
| Zero fruit/vegetable consumption | 0.0 (0) | 0.0 (0) | -- |
| Sweet beverage consumption | 70.5 (122) | 58.2 (178) | χ^2^ (1, N=479)=1.0, p=0.440 |
| Unhealthy/ultra processed food consumption | 86.1 (149) | 71.6 (219) | χ^2^ (1, N=479)=14.3, p=0.005 |
| ^1^Medians compared using Mann Whitney U tests and proportions compared using cluster-adjusted Pearson's χ2 tests.  ^2^Among female adolescents only: urban n=100; rural n=189 | | | |
